# Experimental Design–Guided Optimization of Pervaporative Dehydration of an Esterification Mixture

**DOI:** 10.3390/membranes16070247

**Published:** 2026-07-18

**Authors:** Fatimatou Toure Lo, Magalie Claeys-Bruno, Philippe Moulin, Emilie Carretier

**Affiliations:** 1 Aix Marseille Univ., CNRS, Centrale Med., M2P2 UMR 7340, Equipe Procédés Membranaires (EPM), Europôle de L’arbois, BP80, Pavillon Laennec, Hall C, 13545 Aix en Provence Cedex, France; fatimatou-toure.lo@etu.univ-amu.fr (F.T.L.); emilie.carretier@univ-amu.fr (E.C.); 2 CNRS, IRD, IMBE, Avignon Université, Aix-Marseille University, 13013 Marseille, France; m.claeys-bruno@univ-amu.fr

**Keywords:** esterification, pervaporation, water removal, experimental design, optimization

## Abstract

This study investigates the pervaporation dehydration of a quaternary esterification mixture containing water, 2-ethylhexyl acrylate, 2-ethylhexanol, and propionic acid using a pilot-scale HybSi membrane (BTESE on Al_2_O_3_). A design of experiments was implemented to evaluate the influence of mixture composition on water content in the retentate, permeation flux, and water removal efficiency. Descriptive analysis revealed that the initial water content is the dominant factor governing both permeation flux and dehydration performance, whereas acid, alcohol, and ester have secondary but interactive effects. Reduced cubic polynomial models including linear, binary, and ternary interactions were developed, showing good agreement with experimental data. Ternary diagrams highlighted composition regions where molecular interactions significantly affect separation performance. Multi-response optimization based on desirability functions identified optimal operating conditions at high initial water content (1.146 wt.%), yielding a permeation flux of 0.144 kg·m^−2^·h^−1^, a final water content close to the industrial target (0.2 wt.%), and a water removal efficiency of 86%. Experimental validation confirmed the reliability of the predictive model. The results provide insights into composition–performance relationships and demonstrate the suitability of BTESE membranes for low-water-content esterification systems.

## 1. Introduction

Esterification is a reaction widely used in industry in many fields, including paints, coatings, adhesives, and polymers. Among these compounds, acrylic acid and its esters are particularly important due to their chemical properties and industrial applications. The esterification of acrylic acid with 2-ethylhexanol produces 2-ethylhexyl acrylate, an ester commonly used as a monomer in the formulation of polymers and coatings. However, as with most esterification reactions, the conversion is limited by the presence of water, which is produced during the reaction. Even at low concentrations, water affects both the reaction kinetics and thermodynamics. According to the principle of “Le Chatelier”, an increase in water concentration shifts the equilibrium towards the reactants, reducing ester yield. Water can reduce also catalytic activity. For example, during the esterification of acetic acid with methanol, a decrease in catalytic activity of up to 90% has been observed in the presence of water [1]. Moreover, water promotes side reactions such as hydrolysis (the reverse of esterification) or ester polymerisation, which can affect the quality and reusability of the final product [2]. In mostly organic and hydrophobic systems, water tends to separate from the organic phase, which can cause local accumulations that dilute the reactants and reduce selectivity, making ester purification more difficult. Therefore, the continuous removal of water is often considered a necessary step to enhance esterification performance [3]. Several conventional dehydration processes have been reported in the literature, such as distillation, reactive distillation and reactive extraction [4]. Distillation, usually placed at the reactor outlet, allows the separation of the ester from water and by-products. Reactive distillation, particularly effective for short-chain esters, combines reaction and separation based on differences in volatility between compounds [5]. Reactive extraction, on the other hand, relies on the immiscibility between ester and water, often in the presence of a solvent that also acts as a catalyst [6]. In the case of 2-ethylhexyl acrylate production catalysed by sulfuric acid, the water content in the reaction mixture reached approximately 2.8 wt.% after the reaction. Different configurations have been studied for water removal [7]: flash decantation removed 96.1% of the water, decantation alone removed 96.7% and distillation followed by decantation achieved 99.8% removal. Reactive distillation, which removes water simultaneously with the reaction, also demonstrated its effectiveness. In the production of 2-ethylhexyl acrylate, the batch reaction alone reached a limited conversion of about 30% due to the presence of the acrylic acid-water azeotrope. In contrast, reactive distillation enabled continuous water removal, increasing the reaction conversion to about 80% [8]. However, this process remains complex to implement because it requires precise adjustment of temperature profiles, internal flowrates, and catalyst characteristics to ensure compatibility between reaction conditions and distillation requirements. Reactive extraction has also been applied, using a solvent that acts as a catalyst to increase the conversion rate. Experiments conducted between 60 and 100 °C showed that at 80 °C, ester conversion could reach approximately 85% [6]. However, recycling the catalyst is challenging, as it requires additional separation and extraction steps, increasing operational complexity and costs. In this context, pervaporation is an attractive alternative to conventional dehydration processes. This membrane process allows selective water removal at moderate temperatures, without the use of solvents, while reducing thermal constraints on sensitive compounds. Many studies have shown the benefit of coupling esterification with pervaporation. For example, in isobutyl propionate production, the use of a hydrophilic PVA (polyvinyl alcohol)-PES (polyethersulfone) membrane increased conversion up to 88% at 80 °C, compared with about 70% without pervaporation [9]. For lactic acid esterification, PVA and PAN (polyacrylonitrile) membranes allowed 90–99% of water to be recovered in the permeate at temperatures between 40 and 80 °C; but performance decreased when the water mass fraction in the retentate was below 2% [10]. Similar results were reported for the dehydration of mixtures from ethyl acrylate production using hybrid polymeric and zeolitic membranes at temperatures between 65 and 75 °C, with about 96–99% of the water recovered in the permeate [11]. Despite these promising results, dehydration at very low water contents remains an important challenge for pervaporation. When the water mass fraction decreases below about 2%, process performance decreases, which limits overall efficiency. This issue is particularly important for acrylate-based mixtures. Although permeates with high water content can be obtained, complete dehydration of the reaction mixture remains difficult because fluxes decrease at low water concentrations. To the best of our knowledge, few studies have evaluated the performance of HybSi membranes for the dehydration of quaternary mixtures representative of acrylate synthesis at water contents below a few weight percent, even though these conditions are among the most critical from an industrial point of view.

In this context, this study investigates the dehydration of the reaction mixture from the esterification of acrylic acid with 2-ethylhexanol using pervaporation. The initial mixture contains about 1 wt.% water, and the target is to reduce the water content below 0.2 wt.%. Experiments are carried out at semi-industrial scale using an experimental design approach to evaluate the effect of mixture composition on pervaporation performance. This work aims to improve understanding of pervaporation at very low water contents and to provide useful information for industrial scale-up.

## 2. Materials and Methods

### 2.1. Chemical Products

The mixture from the esterification reaction typically contains four components: acrylic acid (AA), 2-ethylhexanol (2-EH), 2-ethylhexyl acrylate (2-EHA), and water (H_2_O). However, acrylic acid tends to polymerise at high temperatures, so it was replaced by propionic acid, for this study with a synthetic solution, which has similar physicochemical properties. 2-ethylhexyl acrylate (CAS: 103-11-7), 2-ethylhexanol (CAS: 104-76-7) and propionic acid (CAS: 79-09-4) were obtained from Fisher Scientific (Hampton, NY, USA). The water used in this study was purified using a PURELAB^®^ flex 3 system (Elga Lab water; High Wycombe, UK).

### 2.2. Membrane

The ceramic membrane is a multi-channel (19) tubular hydrophilic HybSi membrane [12]: active layer of bis(triethoxysilyl)ethane (BTESE). The membrane length is 1178 mm, the outer diameter is 25 mm, and the membrane area is 0.25 m^2^. The diameter of each channel is 3.5 mm. The membrane is made of three support layers. The final active layer: HybSi has a thickness of 200 to 500 nm and an approximate pore size of 0.4 nm.

### 2.3. Pervaporation Process

**Figure 1:**
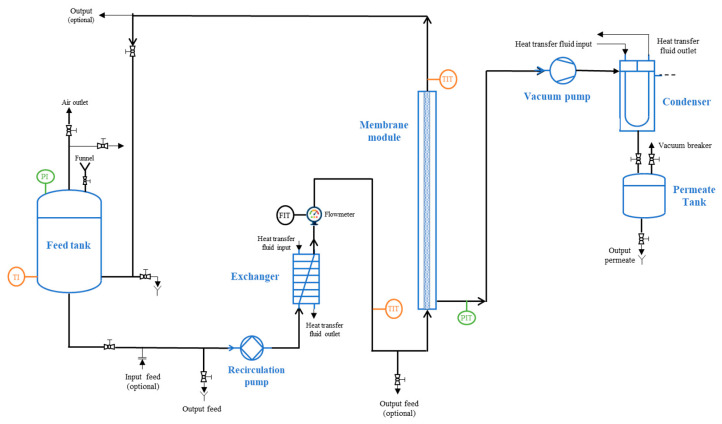
Schematic diagram of semi-industrial pilot unit of pervaporation (TI, PI, temperature and pressure indicator, TIT, PIT, temperature and pressure indicator and controller).

Pervaporation experiments were carried out using a semi-industrial pilot unit operating in batch mode (Figure 1). The system consists of a closed 10 L feed tank made of 316L stainless steel, equipped with pressure and temperature sensors (dial pressure gauge and needle thermometer) to allow accurate monitoring of the conditions inside the tank. For filling, the tank is fitted with a funnel equipped with a valve and an air purge valve. A horizontal centrifugal recirculation pump ensures continuous circulation of the mixture, with a maximum flowrate of 1.2 m^3^ h^−1^ at a pressure of 1 bar. The pump is made of 316L stainless steel and PTFE. A heating unit, located upstream of the membrane, is used to heat the mixture and to maintain the temperature. This unit also includes a cooling coil, an overheating protection system, and a touchscreen that allows the operating temperature to be set and various operating parameters to be monitored. A scroll vacuum pump installed on the permeate line allows a pressure of 10 mbar to be reached and maintains a vacuum pressure below 50 mbar on the downstream side of the membrane throughout the experiment, with a capacity of 6.2 m^3^ h^−1^. Stable vacuum conditions are ensured, which is essential to promote phase change. The heat exchanger consists of a water-cooled refrigeration unit, a recirculation pump, a heat transfer fluid tank containing monoethylene glycol, and a display providing digital temperature readout. The cooling unit operates over a temperature range of −10 to 40 °C and supplies a tubular condenser with an area of 0.2 m^2^. The condenser allows the permeate to be recovered in liquid form and collected in a closed 2L 316L stainless steel vessel, ensuring clean and efficient permeate collection. All experiments were carried out at 80 °C, corresponding to the outlet temperature of the reaction mixture. The volumetric flowrate was set to 0.7 m^3^ h^−1^ to achieve turbulent flow in the membrane and ensure temperature homogeneity. For each experiment run, 3 kg of the quaternary mixture were freshly prepared. The prepared mixture was introduced into the feed tank and heated under recirculation to 80 °C. Once the target temperature was reached, the exact initial composition was determined prior to each experiment by Karl Fischer titration for water content and gas chromatography for organic compounds and after the vacuum pump was switched on, marking the beginning of the pervaporation step. Each experiment was conducted for 1 h: the total duration of the experiment is 3 h, with samples taken every hour, which is equivalent to three separate experiments, each lasting one hour. At the end of this period, permeate and retentate samples were collected for analysis. The pilot unit was then completely drained. To prevent cross-contamination between successive experiments, the entire circuit was thoroughly cleaned with alcohol and flushed before the next run. To assess membrane stability and exclude possible aging effects during the experimental campaign, alcohol permeability was measured before and after process. The ethanol permeation flux was 0.16 kg·m^−2^·h^−1^, and no significant variation was observed. This indicates that no significant change in membrane transport properties was observed under the investigated operating conditions.

To further evaluate the selectivity of the membrane toward water, additional permeation tests were carried out with pure compounds representative of the organic phase. Experiments with pure 2-ethylhexanol and pure 2-ethylhexyl acrylate were performed at 80 °C for a total duration of 3 h, with permeate sampling every 30 min until steady-state flux is reached. The stabilized permeate fluxes were 0.003 kg m^−2^ h^−1^ for 2-ethylhexanol and 0.0016 kg·m^−2^·h^−1^ for 2-ethylhexyl acrylate. These values are significantly lower than the water permeation flux (approximately 12 kg·m^−2^·h^−1^), confirming the strong selectivity of the membrane toward water.

The water removal efficiency is calculated using the following expression:

$$\eta =\;\frac{{water\;mass\;}_{initial}-{water\;mass\;}_{final}}{{water\;mass\;}_{initial}}=\;\frac{m_{feed}\;x_{water}^{feed}-\;m_{retentate}\;x_{water}^{retentate}}{m_{feed}\;x_{water}^{feed}}$$

m_feed_ is the total mass of the feed (g)
x_water_^feed^ is the water mass fraction in the feed
m_retentate_ is the total mass of the retentate (g)
x_water_^retentate^ is the water mass fraction in the retentate

### 2.4. Analytical Methods

The water content in the feed, permeate, and retentate was determined by coulometric Karl Fischer using a Mettler Toledo C10S apparatus (Mettler-Toledo; Zaventem, Belgium) and Hydrana reagent from Honeywell Fluka (Seelze, Germany). The precision of this apparatus given by the manufacturer is between 0.001 and 100%. The concentrations of propionic acid, 2-ethylhexanol, and 2-ethylhexyl acrylate were measured by gas chromatography using Agilent Technologies (Santa Clara, CA, USA) 7890B system equipped with a flame ionization detector (FID) and hydrogen as the carrier gas. An Agilent HP5 column (30 m length, 0.25 µm film thickness, 0.32 mm internal diameter) was used. Helium served as the carrier gas, with a column flowrate of approximately 3.6 mL min^−1^ and a head pressure of about 100 kPa. The injector temperature was set to 240 °C, and the analysis of each sample took approximately 20 min. Two linear alkanes were used as internal standards: n-octane, with a retention time of approximately 4.38 min, for the quantification of the acid, and n-undecane, with a retention time of 13.43 min, for the quantification of the alcohol and ester. An internal calibration method was applied to improve the accuracy of the results. The retention times of the different compounds are presented in Table 1.

### 2.5. Design of Experiments

A design of experiments (DOE) is a statistical methodology used to efficiently plan and analyse experiments to identify and, importantly, quantify the effect of multiple input variables (factors) on one or more output variables (responses) [13]. Unlike the classical approach, which varies one factor at a time, DOE allows the simultaneous study of several factors, reducing the number of experiments needed and accounting for potential interactions between factors. The main target of DOE is to develop a mathematical model linking the measured responses to the input variables, to predict process performance within the experimental domain and determine the optimal operating conditions. In this study, the objective is to optimise the dehydration of the reaction mixture to achieve a final water mass fraction of 0.2% or less, while maximising the permeation flux. To this end, the mass fraction of different components of the mixture were varied, while the pervaporation operating temperature and duration were kept constant.

#### 2.5.1. Factors and Variations Domains

Considering the nature of the system under study, in which the input variables are the proportions of the ester, alcohol, acid, and water, which are interdependent and sum to 1, a mixture design of experiments was selected. This type of design is particularly suited for the study and optimisation of complex formulations, such as chemical and pharmaceutical fields [14]. All input variables represent measurable proportions that can vary over a fixed range. The variation ranges were established by considering both the physicochemical properties of the mixture, particularly conditions close to reaction equilibrium, and the operating conditions of the esterification process. The minimum and maximum limits of each component were defined to ensure the formation of a monophasic mixture, compatible with the pervaporation process (Table 2), and consistent with values typically encountered under industrial conditions. The equilibrium constants of the mixtures used in this experimental design were calculated with values ranging between 0.3 and 3.5. These values fall within the typical range (10^−3^–10^3^) for which a general reaction is at equilibrium, ensuring that the selected conditions are consistent with equilibrium considerations [15].

The influence of mixture composition on the final water content in the retentate (Y_1_) and on the permeation flux (Y_2_) was described using a Scheffe-type polynomial model [16]. A reduced cubic mixture model was selected, considering the linear effects of the components as well as their binary and ternary interactions. The mathematical expression of the model is given by the following equation:

Y_i_ = a_1_X_1_ + a_2_X_2_ + a_3_X_3_ + a_4_X_4_ + b_12_X_1_X_2_ + b_13_X_1_X_3_ + b_14_X_1_X_4_ + b_23_X_2_X_3_ + b_24_X_2_X_4_ + b_34_X_3_X_4_ + c_123_X_1_X_2_X_3_ + c_124_X_1_X_2_X_4_ + c_134_X_1_X_3_X_4_ + c_234_X_2_X_3_X_4_

where Y_i_ is the final water mass fraction in the retentate (Y_1_) or the permeation flux (Y_2_), a_i_ are the coefficients of the linear effects, b_ij_ are the coefficients of the binary interactions, and c_ijk_ are the coefficients of the ternary interactions. The variables X_1_, X_2_, X_3_, and X_4_ represent the proportions of water, 2-ethylhexyl acrylate, 2-ethylhexanol, and propionic acid, respectively.

To estimate the coefficients of the mathematical model, 33 experiments were carefully chosen (D-optimal criteria) (Table 3—in bold). Each run was carried out over a total duration of three hours, with hourly sampling of the permeate and retentate. The compositions measured at each sampling time were used as input conditions for predicting the responses at the subsequent time, which allowed a total of 99 experimental conditions distributed over three-time levels to be exploited.

#### 2.5.2. Desirability

**Figure 2:**
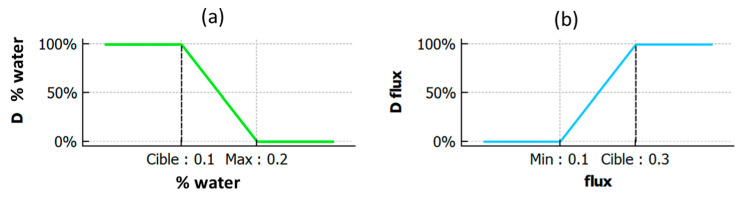
Desirability—preferences: (**a**) minimization of % water and (**b**) maximization of permeation flux.

Process optimization was performed using a multi-response desirability function (Figure 2). Two criteria were considered simultaneously: (i) the water content in the final mixture (Y_1_), and (ii) the permeation flux (Y_2_). Both responses were treated using target-type desirability functions. The target value for the water content was set at 0.2%, while the target for the permeation flux was defined based on the experiment exhibiting the highest flux, to identify an optimal compromise. The responses Y_1_ and Y_2_ were transformed into a dimensionless desirability functions d_i_ ranging from 0 to 100%. The global desirability *D*, given in following equation is a combination of the two *d_i_* values weighted by a coefficient *w*_*i*_. The aim of this coefficient is to give to one or more parameters a higher interest rate than the others in this work, *w*_*i*_ = 1, all the two responses Y_1_ and Y_2_ have the same importance.

$$D={\left(d_{1}^{w_{1}}\times d_{2}^{w_{2}}\right)}^{\frac{1}{\Sigma w_{i}}}$$

When an undesirable value is obtained for at least one of the responses, the global desirability D is 0 and no compromise is achieved. Conversely, when each criterion is fully satisfied, the desirability D reaches 100%. Intermediate values between 0 and 100% indicate an acceptable compromise between the two different responses.

## 3. Results and Discussions

### 3.1. Descriptive Analysis of the Experimental Results

To facilitate the analysis of the experimental before to model and to identify overall trends, the experiments were grouped into several classes of mass fractions for each component of the mixture. The initial water content was divided into intervals corresponding to low, intermediate, and high levels. Similarly, the mass fractions of ester, alcohol, and acid were classified into different levels including the entire investigated composition range. This approach allows easier comparison of process performance for different feed compositions and enables evaluation of the relative influence of each component on the final water content in the retentate, the permeation flux, and the dehydration efficiency. The selected mass fraction classes for each component of the mixture are summarized in Table 4.

#### 3.1.1. Influence of Mixture Composition on Final Water Content and Removal Efficiency

**Figure 3:**
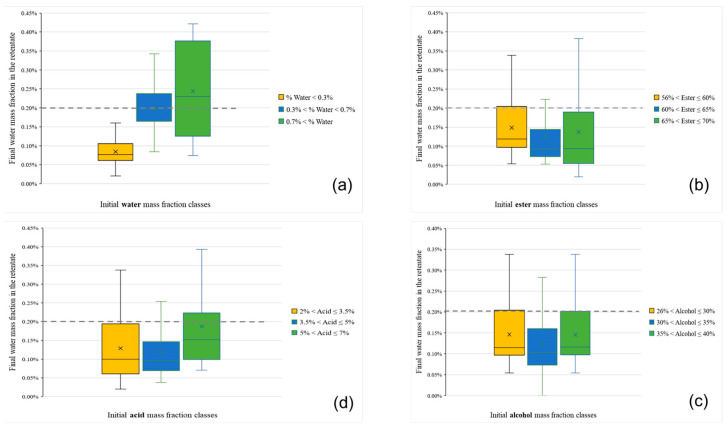
Water content in the retentate as a function of the mass fraction classes of the different components: (**a**) water, (**b**) ester, (**c**) alcohol, and (**d**) acid [duration = 1 h].

Analysis of the results shown in Figure 3 indicates that the final water content in the retentate measured after 1 h of pervaporation does not depend on a single component of the mixture taken individually but rather results from a combined effect of the different components. The target value of 0.2 wt.% can be achieved for all ester, alcohol, and acid mass fraction classes. However, the influence of these variables is not equivalent. Figure 3a shows that the initial water content is the most influential parameter. Indeed, the final water content varies more significantly between the different classes of initial water content than for the other components. At a fixed processing time (1 h), mixtures with higher initial water content exhibit the highest water removal efficiencies (70–90%), due to a larger driving force. However, this does not necessarily imply a lower water content. Even if a large fraction of water is removed, the initially high amount of water may result in a final concentration that remains above the target value (0.2 wt.%). After the initial water content, the acid fraction appears to be the second factor influencing the final water content, as shown in Figure 3d. Intermediate initial acid contents, between 3.5 and 5%, allow lower final water contents to be achieved, in the range of 0.05 to 0.15 wt.%. This effect may be attributed to interactions between the acid and water, as well as interactions between the acid and the membrane, which can influence water sorption and transport. However, ester and alcohol do not appear to show a determining role in reducing the final water content (Figure 3b,c). Their influence appears limited and mainly indirect. It should also be noted that, since the system corresponds to a mixture with a constant total mass fraction, any increase in water content necessarily results in a simultaneous decrease in the mass fractions of ester, alcohol, and acid, which may partly account for the observed trends. With a longer treatment time, these mixtures could probably reach the target value of 0.2 wt.%. As the water content decreases, the process becomes progressively limited by water availability, rather than only by driving force associated with its activity. Thus, at short dehydration times, the initial water content and the acid fraction mainly control the final water content, while alcohol and ester play a more secondary role.

#### 3.1.2. Influence of Mixture Composition on Permeation Flux

**Figure 4:**
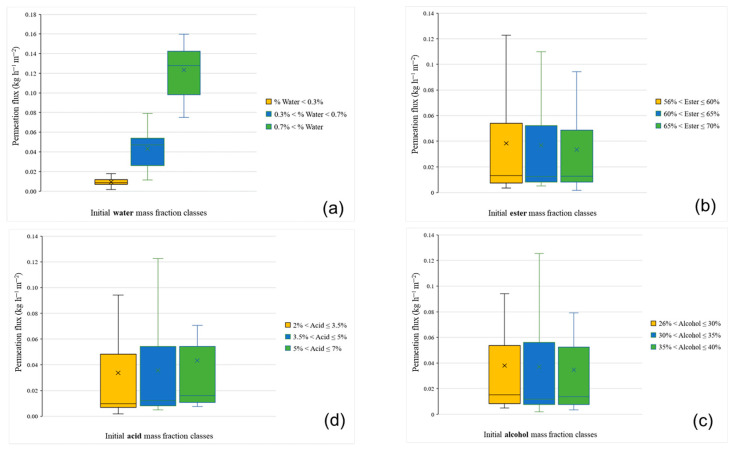
Permeation flux as a function of the mass fraction classes of the different components: (**a**) water, (**b**) ester, (**c**) alcohol, and (**d**) acid [duration = 1 h].

The overall results show that the permeation flux during dehydration is primarily governed by the mixture composition, and more specifically by the water content (Figure 4). As illustrated in Figure 4a, water appears as the dominant factor controlling the permeation flux. As the water mass fraction increases, the flux increases significantly. Although its mass fraction in the feed remains low, ranging between 0.4 and 1 wt.%, the permeation flux can vary by more than one order of magnitude, from values below 0.02 kg·m^−2^·h^−1^ at low water contents to values close to 0.15–0.16 kg·m^−2^·h^−1^ for the mixtures richest in water. This behavior can be explained by the strong affinity of water for the membrane used, which promotes its sorption and diffusion within the BTESE active layer. In this case of hydrophilic membranes, water is preferentially sorbed and diffuses faster than organic compounds, which explains its predominant role in the permeation mechanism during dehydration [17]. This interpretation is further supported by the analysis of the permeate composition: for all initial water content classes investigated, the mass fraction of water in the permeate ranges between 96 and 99%. Three examples are presented in Table 5. The presence of organic compounds in the permeate was almost weak (approximately 1–4%). This behavior can be attributed to the low real partial vapor pressure of each compound at 80 °C, which remains below the applied vacuum pressure (15 mbar). After the initial water content, the acid fraction appears to be the second factor influencing the permeation flux, as shown in Figure 4d. An increase in its mass fraction is associated with a minor increase in the permeate flux. However, the maximum flux does not exceed 0.06 kg·m^−2^·h^−1^, indicating that its influence remains limited. The acid can therefore be considered a secondary component in the dehydration process. In contrast, alcohol and ester do not appear to significantly affect the dehydration performance (Figure 4b,c). Variations in their initial mass fractions only lead to very limited changes in the permeation flux, which remains nearly constant overall. This behavior is consistent with the nature of these compounds, which are weakly polar and have relatively high molar masses. As a result, they exhibit low affinity for the hydrophilic membrane and low permeability through it. The calculated separation factors range from approximately 6800 to 136,000, confirming the very high selectivity of the BTESE membrane toward water.

In summary, the results show that the initial water content of the mixture shows a key role in both the permeation flux and the water removal efficiency. In contrast, the acid, the alcohol, and ester have a more limited and secondary influence. The acid, however, appears to affect the mass transfer within a composition range of 3.5–5% wt.%. The dispersion observed for some compositions therefore suggests the existence of combined effects between the different components of the mixture. Although these preliminary analyses provide useful insights, they do not allow a quantitative assessment of the individual impact of each operating parameter, nor do they enable precise identification of the interactions between variables. To further improve the understanding of the system and to structure the analysis, a modeling approach based on a design of experiments was therefore implemented in the subsequent part of this work.

### 3.2. Modeling Based on Design of Experiments

#### 3.2.1. Modeling of the Water Content in the Retentate (Y_1_)

##### Model Fitting and Statistical Validation (Y_1_)

Based on the experimental results, the coefficients associated with the linear (a_i_), binary (b_ij_), and ternary (c_ijk_) interaction terms were determined by least squares regression. All coefficients expressed in pseudo-components were used to establish the equation relating the final water content in the retentate to the initial mass fractions of each component in the mixture:

Y_1_ = −121.594 X_1_ − 0.028 X_2_ − 0.022 X_3_ − 1.763 X_4_ + 138.791 X_1_X_2_ + 139.782 X_1_X_3_ + 464.665 X_1_X_4_ + 0.0565 X_2_X_3_ + 2.578 X_2_X_4_ + 2.535 X_3_X_4_ − 14.191 X_1_X_2_X_3_ − 461.755 X_1_X_2_X_4_ − 461.528 X_1_X_3_X_4_ + 0.574 X_2_X_3_X_4_

The statistical indicators show that the model exhibits a very good fit to the experimental data, with an R^2^ value of 0.948 (adjusted R^2^ = 0.81). These values indicate a strong consistency between the experimental results and the values predicted by the model.

The model for water content (Y_1_) is further validated using analysis of variance. The regression mean square is 0.0492, while the residual mean square is 0.0014, leading to a Fisher F-value of 35.00. The very low *p*-value (*p* < 0.0001) confirms that the model is statistically significant, and that the variation in the response is mainly explained by the input variables rather than experimental error.

##### Graphical Representation of the Model (Y_1_)

The model equation was used to represent the results in the 3D forms. Each section corresponds to a specific initial water content and is associated with a ternary diagram. In these diagrams, the three axes represent the initial compositions of acid (left axis), alcohol (horizontal axis), and ester (right axis), respectively. This graphical representation is consistently applied to all results. Variations in the final water content are illustrated using a colour scale, with low values shown in blue and high values shown in red, according to the legend.

**Figure 5:**
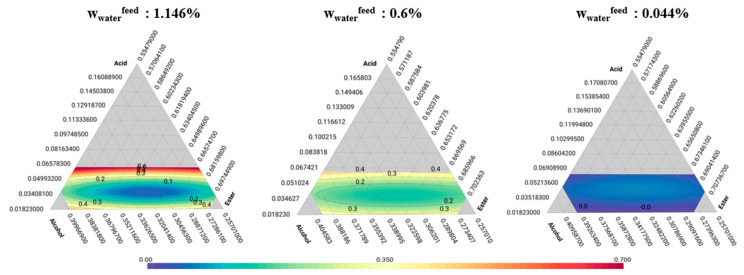
Ternary diagrams showing the variation in final water content as a function of mixture composition, for three levels of water contents [duration = 1 h].

As previously discussed in the descriptive analysis of the experimental results, the ternary diagrams presented in Figure 5 make it possible to identify and quantify composition domains where interactions between components are more pronounced. At high initial water content (1.146%), the final water content in the retentate varies strongly depending on the mixture composition. Under these conditions, the driving force for water transport is high, which promotes water diffusion and evaporation through the membrane. However, a pronounced effect of the acid content can be observed. When the acid mass exceeds 5%, the final water content remains high (≈0.6%), corresponding to a limited water removal efficiency of about 20%. This behavior can be explained by competition between water and acid molecules for the hydrophilic sorption sites of the membrane. Acid molecules may interact with these sites through hydrogen bonding, thereby competing with water for site occupation and reducing both water sorption and water flux through the membrane [18]. In contrast, for acid contents between 3.5 and 5%, the final water content decreases significantly and can reach values as low as 0.1%, corresponding to water removal efficiencies better than or equal to 90%, including high dehydration performance. This behavior is consistent with results reported in the literature. Indeed, dehydration at 75 °C of an aqueous solution containing 90 wt.% isopropanol using a BTESE membrane has been shown to recover approximately 98.9% of water in the permeate [18]. Similarly, another study on the dehydration of a 90 wt.% acetic acid solution using a BTESE membrane at 75 °C reported about 97% water in the permeate; however, a decrease in water and acid permeance was observed as the acid concentration increased [19]. These performances can be explained by the presence of a strong driving force associated with the high initial water content, resulting in high water activity in the mixture. Although the present study is conducted at even lower water contents, the results remain comparable to those reported in the literature, as the water content in the permeate is of the same order of level. This efficiency is also related to the strong affinity of water, a highly polar molecule, for silanol groups and hydrophilic sites within the membrane [19]. Regarding ester and alcohol, their influence remains limited under these high-water-content conditions. Alcohol, which exhibits intermediate polarity, can interact with water; however, its alkyl chain reduces its affinity for the hydrophilic sites of the membrane. Ester molecules, which are less polar, exhibit even weaker specific interactions. Consequently, when water is important in the system, its behavior dominates the dehydration process and tends to mask the effects of these two components. This observation is consistent with pervaporation studies using BTESE membrane, which show that performance is mainly governed by water availability and water-polar compound interactions, whereas less polar organic compounds such as butanol or isopropanol have a moderate effect [20]. At intermediate initial water content (0.6%), the differences related to mixture composition become less pronounced. The final water content becomes more homogeneous, ranging between 0.2 and 0.4 wt.%, while the water removal efficiency also lies within an intermediate range (50–70%). This evolution can be attributed to a reduction in the difference in water activity between the feed and the permeate, leading to a lower driving force for water transport through the membrane. Similar trends have been reported in the literature for the dehydration of bioethanol using hydrophilic composite PVA membranes. In these studies, a final water content of 0.1% could be achieved from a mixture initially containing 5% water; however, the water recovery rate in the permeate progressively decreased as the water content in the retentate decreased, from approximately 99% to 90% for initial water mass fractions of 5% and 1%, respectively. These results confirm that, at intermediate water contents, the decrease in water activity progressively limits dehydration efficiency [21]. At this stage, the effect of the acid is still present but becomes less determining than under high-water-content conditions. At very low initial water content (0.044%), the final water content remains low, with dehydration efficiencies ranging between 20 and 50%. In these conditions, the process becomes mainly limited by water availability, as the driving force is insufficient to enable further dehydration, rather than by the intrinsic properties of the membrane [22].

Although the ternary diagrams were analyzed separately to better identify the influence of each component on dehydration performance, it is also important to discuss the results following the actual progression of a dehydration experiment, starting from high initial water content, and approaching the target value of 0.2 wt.%. A general trend can be observed. When the water content is high, the mass transfer driving force is strong, and the mixture composition, particularly the acid content, significantly influences dehydration performance. As the water content decreases, this effect progressively weakens because the difference in water activity between the feed and the permeate becomes smaller. Finally, near the target water content (0.2 wt.%), water availability becomes the main limiting factor of the process, and the influence of mixture composition becomes secondary. Alcohol and ester show only a weak, almost negligible, influence throughout the dehydration process, as their interactions with the membrane are less pronounced than those of water and acid.

#### 3.2.2. Modeling of the Permeation Flux (Y_2_)

##### Model Fitting and Statistical Validation (Y_2_)

Similarly, to the modelling of the water content, the set of estimated coefficients was used to establish the following equation relating the permeation flux to the initial mass fractions of water, alcohol, acid, and ester expressed in pseudo-components:

Y_2_ = 6.178 X_1_ − 0.004 X_2_ − 0.014 X_3_ − 0.851 X_4_ − 4.186 X_1_X_2_ − 3.858 X_1_X_3_ + 35.8267 X_1_X_4_ − 0.007 X_2_X_3_ + 1.125 X_2_X_4_ + 1.152 X_3_X_4_ + 1.197 X_1_X_2_X_3_ − 54.244 X_1_X_2_X_4_ − 52.193 X_1_X_3_X_4_ − 0.184 X_2_X_3_X_4_

The model for permeation flux (Y_2_) also shows a very good agreement with the experimental data, with an adjusted R^2^ value of 0.94.

The analysis of variance results indicates a strong statistical significance of the model. The regression mean square is 0.01356, while the residual mean square is 0.00011, resulting in a high Fisher F-value of 126.06. The *p*-value (*p* < 0.0001) confirms that the model is highly significant, meaning that the variation in permeation flux is mainly governed by the input variables rather than experimental error.

##### Graphical Representation of the Model (Y_2_)

**Figure 6:**
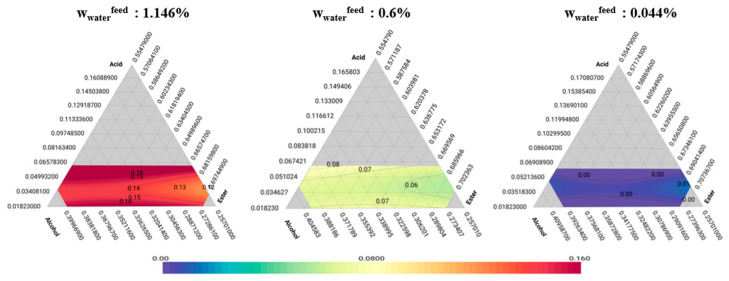
Ternary diagrams showing the variation in permeation flux as a function of mixture composition, for three levels of water contents [duration = 1 h].

As for the water content, the model equation was also used to generate the ternary diagrams presented in Figure 6.

The permeation flux observed in the ternary diagrams varies significantly with the mixture composition. At high initial water content (1.146%), the flux reaches values between 0.13 and 0.15 kg·m^−2^·h^−1^ for acid mass fractions ranging from 3.5 to 5%. This region corresponds to an equilibrium between high water activity and still limited competition of organic compounds for the hydrophilic sites of the membrane. This comportment is consistent with classical pervaporation theory, according to which the permeation flux is proportional to the chemical potential gradient [17]. Moreover, several studies using BTESE membranes have shown that increasing water content leads to an increase in permeation flux due to enhanced water sorption within the organosilica network [23,24]. Although high acid concentrations (≈5%) generally tend to reduce water permeation, relatively high fluxes (≈0.16 kg·m^−2^·h^−1^) were still observed. This can be explained by the dominance of the driving force associated with high water activity over water-acid or acid-membrane interaction effects. When the acid content decreases below 5%, corresponding to an optimal range, water-acid competition becomes more significant, and the permeation flux begins to slightly decrease. At intermediate initial water content (0.6%), the permeation flux decreases significantly and mostly ranges between 0.06 and 0.08 kg·m^−2^·h^−1^. This reduction can be attributed to a decrease in water activity, leading to a lower driving force. Similar trends have been reported for the dehydration of binary water/alcohol or water/acid mixtures, where reduced water activity directly limits water sorption and, consequently, the permeation flux [25,26]. Finally, at very low water mass fractions (0.044%), the permeation flux becomes almost negligible (<0.01 kg·m^−2^·h^−1^). In this regime, the process is no longer governed by the intrinsic properties of the membrane but rather by the limited availability of water.

A comparison with literature data (Table 6) shows that the permeation fluxes obtained in this study are of the same order of magnitude as those previously reported, particularly at low initial water contents. The relatively lower values observed here can be attributed to the very low water content (<1%) and to the simultaneous presence of other organic compounds in the mixture, especially acids, in contrast to many studies focusing on binary water/alcohol or water/acid systems with higher water contents.

### 3.3. Multi-Response Optimization: Desirability Function

**Figure 7:**
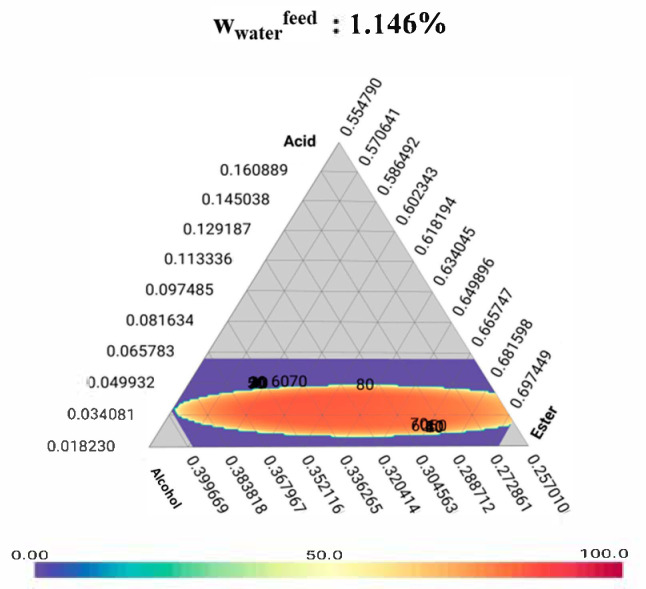
Global desirability ternary diagram of the pervaporation process as a function of mixture composition.

Process optimization was carried out using a multi-response desirability function to identify the operating conditions providing the best overall compromise between several performance criteria. Two responses were simultaneously considered: (i) the residual water content in the final mixture, and (ii) the permeation flux. The two responses were treated using target-type desirability functions. The target value for the residual water content was fixed at 0.2 wt.%, corresponding to industrial specifications. The target associated with the permeation flux was defined based on the experiment that exhibited the maximum measured value (0.3 kg·m^−2^·h^−1^). This value is used as an optimization target, although it is not reached experimentally, and it does not affect the determination of the optimal conditions. The ternary diagram of global desirability D obtained for the highest initial water content is presented in Figure 7.

For high initial water content (1.146%), a broad region of high desirability is observed (orange zone), characterized by values above 80%. In this region, the permeation flux remains high, the final water content remains low, and a satisfactory water removal efficiency is achieved. However, for lower initial water contents (0.6% and 0.044%), the desirability values are very low or negligible. Under these conditions, at least one of the considered criteria is not satisfied, preventing the identification of an optimal operating region. These results confirm that decreasing the initial water content strongly limits the overall process performance. As previously explained, a low initial water mass fraction leads to lower permeation fluxes. Since the desirability function aims to maximize the flux, this directly penalizes the performance criteria. Consequently, the ternary diagrams corresponding to these low water contents show zero desirability values. Therefore, only the diagram for the highest initial water content (1.146%) is presented in Figure 7, as it allows the identification of accurate optimal region favorable for the process. The optimal mixture determined by Azurad software (version 4.6.2.), and its characteristics are presented in Table 7.

### 3.4. Experimental Validation of the Optimal Conditions

To verify the reliability of the optimal conditions predicted by the model, additional experiments were performed at the identified optimal composition. Three independent replicates were conducted to assess the reproducibility of the results. The experimental values obtained for the residual water content, permeation flux, and water removal efficiency were compared with the corresponding model predictions.

The comparison (Table 8) shows a good agreement between predicted and experimental values for permeation flux and final water content. The calculated water removal efficiency is approximately 86% ± 4%. The relative errors remain low, confirming the strong predictive capability of the model under optimal conditions. In contrast, a larger variation is observed for the final water content. This difference can be attributed to the high sensitivity of this response at very low water concentrations, where small absolute variations may result in large relative errors. Moreover, analytical uncertainty becomes more significant in this low concentration range. Despite this variation, the experimentally obtained water content remains close to the targeted industrial specification, indicating that the identified optimal conditions remain operationally important.

## 4. Conclusions

This work investigated the pervaporation dehydration of a quaternary esterification mixture using a HybSi membrane (BTESE on Al_2_O_3_). A statistical mixture design approach was employed to model and analyze the influence of the initial mass fractions of water, acid, ester, and alcohol on three key performance indicators: final water content, permeation flux, and water removal efficiency. The developed regression models showed good predictive performance, with high coefficients of determination and good statistically significant parameters. Ternary diagrams enabled the identification of composition regions where molecular interactions significantly influence process performance. Results demonstrated that the initial water content is the dominant parameter controlling both permeation flux and dehydration efficiency. At high initial water content (1.146%), strong driving force and favorable water-membrane interactions led to high fluxes (up to 0.16 kg m^−2^ h^−1^) and removal efficiencies above 90%. In contrast, at low water content (0.044%), the process was limited by water availability, resulting in very low flux values (<0.01 kg m^−2^ h^−1^). Acid concentration induces competitive sorption effects that may reduce water transport at higher fractions, whereas Alcohol and ester exhibit a secondary influence. Multi-response desirability optimization identified optimal operating conditions at high initial water content (1.146%), yielding a permeation flux of 0.144 kg m^−2^ h^−1^ and a water removal efficiency of 86%. Experimental validation confirmed good agreement with model predictions.

From an industrial perspective, these results also highlight the importance of controlling the acid content at the outlet of the esterification step. Maintaining this content between 3.5 and 5% would help reduce competition with water at the membrane level and consequently enhance the efficiency of the dehydration step by pervaporation.

Further work on binary and ternary systems would help clarify competitive and interaction effects between components in the membrane. And the development of a mechanistic model coupling esterification kinetics and pervaporation mass transfer would be a valuable perspective for future work in the context of an integrated reaction–separation process.

## Figures and Tables

**Table 1:** Retention times of compounds in the column.

	Propionic Acid	n-Octane	2-Ethylhexanol	n-Undecane	2-Ethylhexyl Acrylate
Retention time (min)	3.27	4.38	11.34	13.43	17.22

**Table 2:** Variation ranges of the studied factors.

Variables	Factors	Variation Domains Mass Fraction
X_1_	Initial water content	0.4–1%
X_2_	2-Ethylhexyl acrylate	56–70%
X_3_	2-Ethylhexanol	26–40%
X_4_	Propionic acid	2–7%

**Table 3:** Experimental design.

Test	Water	Ester	Alcohol	Acid	Test	Water	Ester	Alcohol	Acid
**1**	**0.0043**	**0.6745**	**0.2814**	**0.0398**	34	0.0008	0.6742	0.2778	0.0472
**2**	**0.0041**	**0.6772**	**0.2603**	**0.0584**	35	0.0034	0.6783	0.257	0.0613
**3**	**0.0096**	**0.5981**	**0.3664**	**0.0259**	36	0.0025	0.602	0.369	0.0265
**4**	**0.0042**	**0.5997**	**0.3522**	**0.0439**	37	0.002	0.5889	0.3651	0.044
**5**	**0.01**	**0.6045**	**0.3384**	**0.0471**	38	0.0036	0.6103	0.3398	0.0463
**6**	**0.0043**	**0.5741**	**0.3718**	**0.0498**	39	0.0025	0.5768	0.3713	0.0494
**7**	**0.0064**	**0.5921**	**0.3745**	**0.027**	40	0.0019	0.5937	0.3732	0.0312
**8**	**0.007**	**0.6253**	**0.3262**	**0.0415**	41	0.0021	0.6135	0.3406	0.0438
**9**	**0.0038**	**0.6441**	**0.3264**	**0.0257**	42	0.0019	0.648	0.3233	0.0268
**10**	**0.0107**	**0.6194**	**0.3229**	**0.047**	43	0.0014	0.6275	0.324	0.0471
**11**	**0.0071**	**0.6832**	**0.2769**	**0.0328**	44	0.0019	0.6805	0.2802	0.0374
**12**	**0.0105**	**0.6875**	**0.2743**	**0.0277**	45	0.0012	0.7004	0.2737	0.0247
**13**	**0.01**	**0.6862**	**0.2708**	**0.033**	46	0.0018	0.6927	0.272	0.0335
**14**	**0.0101**	**0.621**	**0.3307**	**0.0382**	47	0.0009	0.6299	0.3346	0.0346
**15**	**0.0105**	**0.5684**	**0.3823**	**0.0388**	48	0.001	0.576	0.3857	0.0373
**16**	**0.0041**	**0.5963**	**0.3731**	**0.0265**	49	0.0012	0.5974	0.3737	0.0277
**17**	**0.0104**	**0.6411**	**0.3207**	**0.0278**	50	0.0007	0.6507	0.3233	0.0253
**18**	**0.0041**	**0.6235**	**0.3272**	**0.0452**	51	0.002	0.6225	0.3253	0.0502
**19**	**0.004**	**0.667**	**0.2784**	**0.0506**	52	0.002	0.6695	0.2747	0.0538
**20**	**0.0043**	**0.636**	**0.3226**	**0.0371**	53	0.0017	0.6403	0.3229	0.0351
**21**	**0.0038**	**0.5859**	**0.3899**	**0.0204**	54	0.0028	0.5895	0.3868	0.0209
**22**	**0.0075**	**0.6321**	**0.3401**	**0.0203**	55	0.0026	0.6287	0.3461	0.0226
**23**	**0.0106**	**0.6802**	**0.2885**	**0.0207**	56	0.0042	0.6739	0.3	0.0219
**24**	**0.0106**	**0.6051**	**0.3285**	**0.0558**	57	0.0038	0.6272	0.3198	0.0492
**25**	**0.011**	**0.5607**	**0.3777**	**0.0506**	58	0.0039	0.5675	0.3684	0.0602
**26**	**0.0042**	**0.6781**	**0.2957**	**0.022**	59	0.0024	0.6738	0.3035	0.0203
**27**	**0.0112**	**0.5816**	**0.3832**	**0.024**	60	0.0034	0.5849	0.3835	0.0282
**28**	**0.0035**	**0.6105**	**0.3244**	**0.0616**	61	0.0022	0.6231	0.321	0.0537
**29**	**0.0044**	**0.5545**	**0.3858**	**0.0553**	62	0.0021	0.5751	0.3771	0.0457
**30**	**0.011**	**0.6686**	**0.266**	**0.0544**	63	0.0038	0.6641	0.2804	0.0517
**31**	**0.004**	**0.5856**	**0.3903**	**0.0201**	64	0.0023	0.5884	0.3897	0.0196
**32**	**0.0041**	**0.5763**	**0.3979**	**0.0217**	65	0.0025	0.58	0.3961	0.0214
**33**	**0.0092**	**0.5999**	**0.3488**	**0.0421**	66	0.0019	0.6058	0.3503	0.042
67	0.0005	0.6772	0.2771	0.0452	84	0.0013	0.6252	0.3245	0.049
68	0.0017	0.6818	0.2578	0.0587	85	0.0007	0.6781	0.2721	0.0491
69	0.0011	0.6073	0.3599	0.0317	86	0.001	0.6383	0.3219	0.0388
70	0.0012	0.5899	0.3668	0.0421	87	0.0016	0.5935	0.3844	0.0205
71	0.0009	0.6139	0.3396	0.0456	88	0.0008	0.6338	0.3448	0.0206
72	0.0016	0.5814	0.3732	0.0438	89	0.0024	0.6783	0.2983	0.021
73	0.0011	0.5949	0.3741	0.0299	90	0.0013	0.6281	0.3199	0.0507
74	0.001	0.6159	0.3396	0.0435	91	0.0015	0.5669	0.3683	0.0633
75	0.0009	0.6483	0.3217	0.0291	92	0.0011	0.677	0.3017	0.0202
76	0.0007	0.6303	0.3235	0.0455	93	0.001	0.587	0.384	0.028
77	0.0009	0.6972	0.278	0.0239	94	0.0011	0.6215	0.3199	0.0575
78	0.0006	0.7017	0.2734	0.0243	95	0.0012	0.5723	0.3761	0.0504
79	0.0007	0.6952	0.2713	0.0328	96	0.0017	0.6679	0.2766	0.0538
80	0.0006	0.6311	0.3339	0.0344	97	0.0012	0.5896	0.391	0.0182
81	0.0007	0.5765	0.3839	0.0389	98	0.0011	0.5796	0.3981	0.0212
82	0.0007	0.599	0.3732	0.0271	99	0.0012	0.6097	0.3481	0.041
83	0.0005	0.6519	0.3225	0.0251					

**Table 4:** Proportion classes of each component.

Component	Low	Intermediate	High
Water	<0.3%	0.3–0.7%	0.7–1%
2-Ethylhexyl acrylate	56–60%	60–65%	65–70%
2-Ethylhexanol	26–30%	30–35%	35–40%
Propionic acid	2–3.5%	3.5–5%	5–7%

**Table 5:** Examples of water content in the feed and in the permeate for different pervaporation experiments, [duration = 1 h].

Test No.	Initial Water Content	Water Content in the Permeate
2	0.409%	96.253%
8	0.704%	97.617%
14	1.006%	99.444%

**Table 6:** Comparison of selected dehydration studies as a function of mixture composition and membrane type at temperature ≤ 80 °C.

Reference	Mixture	Membrane	Initial Water Content (%)	Permeation Flux (kg m^−2^ h^−1^)	Comments
[23]	Water/acetic acid	BTESE	≈10	2–4	High water permeation
[27]	Water/alcohols (butanol, IPA)	Hydrophilic silica	≈5	0.1–1	High water selectivity; limited alcohol diffusion
[28]	Water/acid	Zeolite (ZMS-5)	≈10	0.2–0.3	Flux decreases with increasing water content
	Quaternary (esterification)		≈14	0.4–0.63	
[11]	Quaternary (esterification)	Pervap#1201	2–15	0.05–1.2	Flux decreases with increasing water content
[29]	Water/isopropanol	PVA	7–18	0.069–1.0275	Flux decreases with increasing initial water content
[21]	Bioethanol: ethanol (82–89%)/water/methanol (2.3–6.9%)/traces other products	Hydrophilic PVA composite	6–8.5	0.025–0.4	Temperature and initial water content strongly affect performance; flux increases with water content; traces of alcohols detected in the permeate
This study	Quaternary (esterification)	HybSi (BTESE on Al_2_O_3_)	0.044–1.146	0.01–0.16	Negative effect of high acid content on dehydration; flux decreases at low water content

**Table 7:** Characteristics of the optimal operating conditions.

Factor	Value	Response	Predicted Value
Water (X_1_)	1.15%	Global desirability	79.94%
Ester (X_2_)	62.74%	Final water content	0.00104
Alcohol (X_3_)	32.51%	Permeation flux (kg m^−2^ h^−1^)	0.1437
Acid (X_4_)	3.60%		

**Table 8:** Comparison between predicted and experimental responses at the optimal operating conditions.

Response	Predicted Value	Experimental Value
Final water content	0.104 wt.%	0.154% ± 0.040%
Permeation flux (kg m^−2^ h^−1^)	0.1437	0.1409 ± 0.0094

## Data Availability

The data are available on request by email to the corresponding author.
